# A phase II trial for the efficacy of physiotherapy intervention for early-onset hip osteoarthritis: study protocol for a randomised controlled trial

**DOI:** 10.1186/s13063-014-0543-7

**Published:** 2015-01-27

**Authors:** Joanne L Kemp, Kate Moore, Marlene Fransen, Trevor G Russell, Kay M Crossley

**Affiliations:** Australian Centre for Research into Injury in Sport and its Prevention (ACRISP), Federation University Australia, PO Box 663, Ballarat, 3350 VIC Australia; Bodysystem Physiotherapy, 38 Collins St, Hobart, 7000 TAS Australia; School of Physiotherapy, University of Sydney, 75 East St, Lidcombe, Sydney, 2141 NSW Australia; School of Health and Rehabilitation Sciences, University of Queensland, St Lucia, Brisbane, 4067 QLD Australia

**Keywords:** Osteoarthritis, Hip joint, Hip arthroscopy, Randomised controlled trial, Physiotherapy

## Abstract

**Background:**

Early-onset hip osteoarthritis is commonly seen in people undergoing hip arthroscopy and is associated with increased pain, reduced ability to participate in physical activity, reduced quality of life and reduced range of motion and muscle strength. Despite this, the efficacy of non-surgical interventions such as exercise therapies remains unknown. The primary aim is to establish the feasibility of a phase III randomised controlled trial investigating a targeted physiotherapy intervention for people with early-onset hip osteoarthritis. The secondary aims are to determine the size of treatment effects of a physiotherapy intervention, targeted to improve hip joint range and hip-related symptoms in early-onset hip osteoarthritis following hip arthroscopy, compared to a health-education control.

**Methods:**

This protocol describes a randomised, assessor- and participant-blind, controlled clinical trial. We will include 20 participants who are (i) aged between 18 and 50 years; (ii) have undergone hip arthroscopy during the past six to 12 months; (iii) have early-onset hip osteoarthritis (defined as chondrolabral pathology) at the time of hip arthroscopy; and (iv) experience hip-related pain during activities. Primary outcome will be the feasibility of a phase III clinical trial. Secondary outcomes will be (i) perceived global change score; (ii) hip-related symptoms (measured using the Hip disability and Osteoarthritis Outcome Score (HOOS) pain subscale, activity subscale, and sport and recreation subscale); (iii) hip quality of life (measured using the HOOS quality of life subscale and International Hip Outcome tool; (iv) hip muscle strength and (v) hip range of motion. The physiotherapy intervention is semi-standardised, including joint and soft tissue mobilisation and stretching, hip and trunk muscle retraining and functional and activity-specific retraining and education. The control intervention encompasses individualised health education, with the same frequency and duration as the intervention. The trial primary end-point is the conclusion of the 12-week intervention, and follow-up measures will be collected at the 12-week post-baseline assessment.

**Discussion:**

The findings of this study will provide guidance regarding the feasibility of a full-scale phase III randomised controlled trial, prior to its undertaking.

**Trial registration:**

The trial protocol was registered with the Australian Clinical Trials Registry (number: 12614000426684) on 17 April 2014.

**Electronic supplementary material:**

The online version of this article (doi:10.1186/s13063-014-0543-7) contains supplementary material, which is available to authorized users.

## Background

Hip pathology is a common cause of hip pain [1,2], and is associated with considerable morbidity in people aged between 18 and 50 years [3,4]. In recent years, arthroscopic surgery has contributed to advancements in assessment and management of hip pain [5]. Recently, the number of hip arthroscopic procedures performed in the United States [6-8], United Kingdom, Australia [9] and Asia [10] has increased dramatically. In Australia, Medicare data indicate that between 2010 and 2013 the number of people undergoing hip arthroscopy increased by over 50% [9], while in the United States the rate of hip arthroscopic surgery increased six-fold between 2006 and 2010 [7]. Despite good results at between five and 10 years post-arthroscopy years, those with osteoarthritis (OA) at arthroscopy consistently report less favourable outcomes when compared to those without OA [11]. In addition, we recently observed that early-onset hip OA is associated with worse outcomes in people who have undergone hip arthroscopy [12].

Musculoskeletal conditions are second only to mental and behavioural disorders, as global contributors to years lived with disability [13]. Due to its negative impact on individual functioning and health service expenditure, OA has been designated a National Health priority area [14]. The hip joint is a common site for OA [15], affecting approximately 12% of adults in the United Kingdom [16] and the United States of America [17]. As there is no cure for hip OA, the identification of non-surgical interventions that can reduce the progression of hip-related symptoms is important, as this will reduce disease burden [18].

We recently reported that chondrolabral pathology, a marker of early-onset hip OA, is common in people who undergo hip arthroscopy for hip pain, and is associated with worse pain, difficulty participating in physical activity and reduced quality-of-life compared to healthy controls [19]. Moreover, it appears that early onset hip OA has a significant impact on young and middle-aged people being able to participate in physical activities without difficulty, which could ultimately lead to physical inactivity. Inactivity is associated with adverse health outcomes, which include type two diabetes, ischaemic heat disease, stroke, depression and certain cancers [20]. If the progression of hip OA symptoms can be slowed in its early stages, people with hip OA may participate in greater levels of physical activity, limiting the public health burden of this disease [21,22].

A full-scale phase III randomised controlled trial (RCT) is costly, and before undertaking such a study it is important to establish its feasibility [23,24]. In Australia, hip arthroscopy is mostly conducted in the private sector and such patients may be unwilling to participate in an RCT. In addition, no RCT examining the effects of a physiotherapy intervention has been undertaken in this population, and adherence with the intervention is unknown. The treatment algorithm has not been tested within the constraints of a clinical trial and the adverse events are not known. Therefore, before committing to a full-scale RCT, the feasibility of such a study should be established by undertaking a phase II RCT [24].

The primary aim of this study is to establish the feasibility of a phase III RCT investigating a targeted physiotherapy intervention for people with early-onset hip OA. The secondary aims are to determine the size of treatment effects of a physiotherapy intervention, targeted to improve hip joint range and hip-related symptoms in early-onset hip OA following hip arthroscopy, compared to a health-education control.

## Methods/Design

### Experimental design

This protocol describes a randomised, assessor- and participant--blind, controlled clinical trial conforming to Standard Protocol Items: Recommendations for Interventional Trials (SPIRIT) [25] guidelines. The trial protocol was registered with the Australian Clinical Trials Registry (ACTR number: 12614000426684) on 17 April 2014. Ethics approval was obtained through the University of Queensland Medical Research Ethics Committee (number: 2013001553).

### Participants

A total of 20 participants will be recruited through a single orthopedic surgeon (MGP) in Hobart, Australia, with extensive expertise in hip arthroscopy. This number of participants was chosen in order to determine the feasibility of recruitment into a larger scale phase III trial, as we estimate that 20 participants represents 30% of eligible patients from a single surgeon. In addition, 20 participants will allow for observation of sample variability and any possible adverse responses to the intervention. A project investigator (KM) will screen for eligibility based on history and examination.

#### Inclusion criteria

The inclusion criteria are as follows: (i) aged between 18 and 50 years; (ii) arthroscopy for intra-articular hip pathology during the past six to 12 months; (iii) evidence of early-onset hip OA (defined as chondrolabral pathology) at time of hip arthroscopy; (iv) hip-related pain during activities such as sitting, squatting, stair ambulation or twisting on the leg and (v) hip-related pain score of over 30 on a 100 mm visual analogue scale (VAS).

#### Exclusion criteria

The exclusion criteria are as follows: (i) pain that is not confirmed by physical examination of the hip [26,27]; (ii) concurrent symptoms of hip bursitis or tendinitis; (iii) surgical complications, including infection; (iv) planned lower limb surgery in the following 12 months (such as an arthroplasty); (v) physical inability to weight-bear fully or undertake testing procedures and (vi) inability to understand written and spoken English.

### Procedure

**Figure 1:**
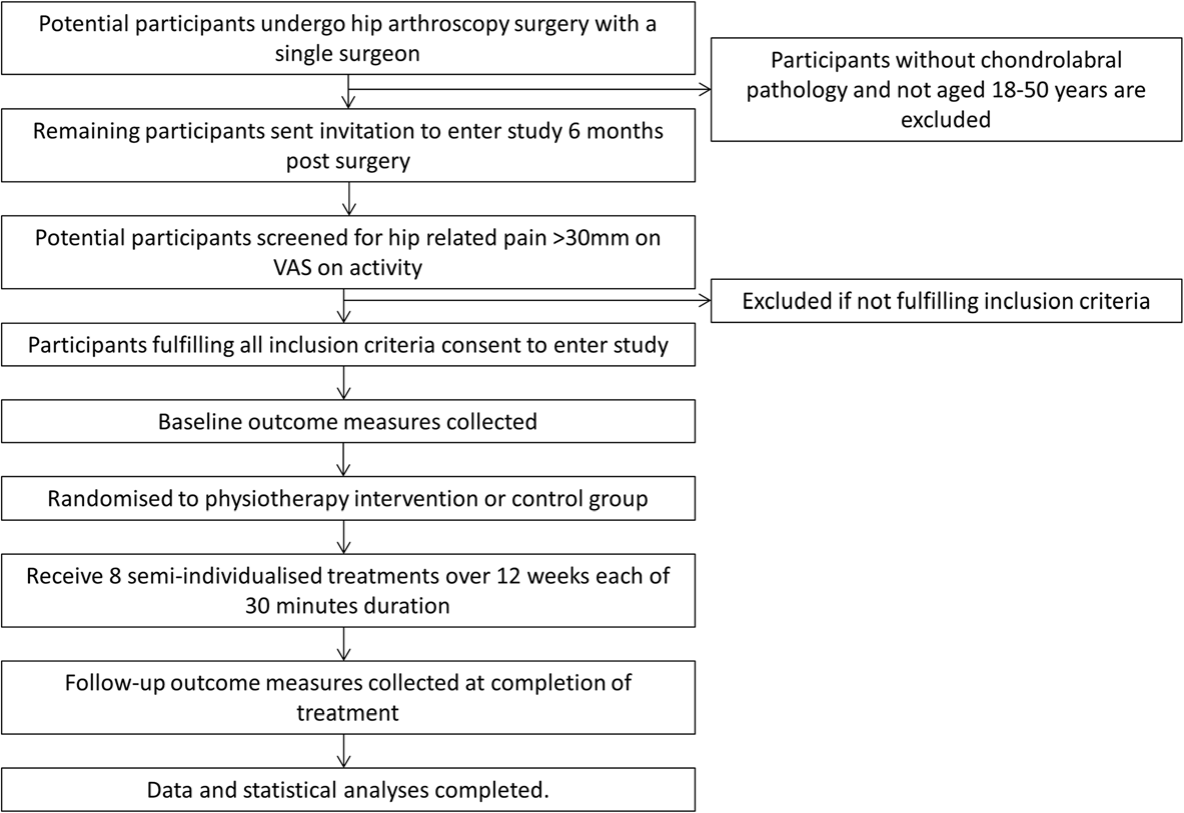
**Flowchart of trial.**

Potential participants will be identified by the surgeon and invited to contact the project coordinator (JK). (Figure 1) The project coordinator will contact potential participants by phone if they do not respond to the initial invitation. Volunteers will be screened via telephone interview, followed by a clinical examination to confirm eligibility (KM). The randomisation schedule will be generated and maintained centrally by the University of Queensland, School of Health and Rehabilitation Sciences, and will be revealed to the project coordinator (JK) via telephone following the baseline assessment. The blinded researcher (KM) will obtain informed consent (Additional file 1) and will perform outcome assessments at baseline and three months. Participants will be instructed not to divulge their group allocation to the assessor. While physiotherapists cannot be blinded to group allocation, participants will be informed that they can receive one of two possible interventions. Thus, participants will remain blinded to treatment allocation. Participants will be asked to refrain from other treatments, but stable drug doses will be permitted. Physiotherapists will record per protocol treatment. Participants will record adherence with home exercises, adverse events and any co-interventions in a log book.

### Outcome assessment

Outcomes will be assessed at baseline and at the trial primary end-point, which is at the conclusion of the 12-week intervention.

### Primary outcome measure: feasibility of a full-scale randomized controlled trial

Feasibility will be assessed by evaluating the willingness of participants to enrol; the number of eligible participants; the recruitment rate; adherence to the intervention, home exercises and log-book completion and the drop-out rate. Adverse events will be recorded.

### Secondary outcome measures: perceived global change score

Participants will rate their perceived change following treatment on a six-point ordinal scale (completely recovered, much improved, improved, no change, worse and much worse) [28]. Measuring patient-perceived change using such scales has been shown to be clinically relevant and a stable concept for interpreting meaningful improvements from an individual perspective [29].

### Secondary outcome measure: hip-related symptoms

The Hip disability and Osteoarthritis Outcome Score HOOS-pain [30] will measure hip-related symptoms. The HOOS was evaluated in younger adults with OA [30], and incorporates the Western Ontario MacMaster Universities Osteoarthritis Index (WOMAC) 3.0LK [31]. The HOOS-pain subscale is equivalent to the WOMAC pain subscale. We have determined that the HOOS-pain subscale is reliable (intraclass correlation coefficient (ICC) = 0.96; 95% confidence interval (CI): 0.93 to 0.98), with a low standard error of measurement (SEM) of four points out of 100, and is valid and responsive, with a minimal clinically important change (MCIC) of nine points [32].

### Secondary outcome measures: hip-related quality of life

The International Hip Outcome tool (iHOT-33) and HOOS quality of life subscale (HOOS-Q) will measure hip-related quality of life. The iHOT-33 is a composite score that was developed for specific use in the hip arthroscopy population [33]. We determined that it is reliable (ICC = 0.93; 95% CI: 0.87 to 0.96), with a low SEM of six points out of 100, and is valid and responsive, with an MCIC of 10 points [32]. The HOOS-Q [30] is reliable (ICC = 0.95; 95% CI: 0.84 to 0.97), with a low SEM of five points out of 100, and is valid and responsive, with an MCIC of 11 points [32].

### Secondary outcome measures: other hip-related symptoms

Other hip-related symptoms will include the HOOS activity of daily living subscale (HOOS-A), the HOOS sport and recreation subscale (HOOS-Sp) and HOOS symptoms and stiffness subscale (HOOS-S). We determined these subscales were reliable (ICC: 0.93 to 0.96), with low SEMs of three to six points, and have MCICs ranging from six to 10 points [34].

### Secondary outcome measures: hip muscle strength and hip joint range

Hip abduction, extension and external rotation strength and hip flexion range will be measured using our previously published methods [35,36], with high reliability (ICC: 0.87 to 0.95). Briefly, all strength tests will be performed with a Commander Power track II (J-Tech medical, Salt Lake City, Utah, USA) hand-held dynamometer. The tester will match the force generated by the participant performing an isometric muscle contraction (the ‘make’ test) [37] and the best of three tests will be recorded. Strength will be recorded as a torque measure, calculated by multiplying the force (measured in Newtons (N)) by the length of the moment arm (measured in metres (m)), and then data will be normalized for body weight (measured in kilograms ((kg) Nm/kg). Specifically, abductor strength will be measured in the supine position, with stabilisation of the contra-lateral thigh, and external rotation strength will be measured in the prone position, with stabilisation of the contra-lateral thigh [35]. Hip flexion range will be measured in the supine position as an active range of motion measure, with stabilisation of the contra-lateral thigh. It will be measured using a Plurimeter inclinometer (Dr Rippstein, La Conversion, Switzerland) as the mean of three measures [36].

### Other measures

#### Potential covariates for statistical analyses

The potential covariates for statistical analyses are body anthropometry: weight, height, body mass index and waist girth.

#### Interventions

Each participant will be treated by experienced physiotherapists who will be trained and proficient in both interventions (physiotherapy and control). The physiotherapists will receive two training sessions prior to the commencement of the study. Monthly meetings between the treating physiotherapists and the project coordinator (JK) will occur throughout the trial to ensure that the physiotherapy intervention remains consistent. The intervention is a face-to-face physiotherapy intervention, which will be delivered in eight sessions over three months (once per week for four weeks, then once per fortnight for eight weeks). This semi-standardised type of intervention has been described previously in an RCT protocol for post-operative physiotherapy in patients with femoro-acetabular impingement [38]. Participants will be asked to refrain from other physiotherapy interventions during the trial. All participants will be able to discuss concerns regarding their condition with the project coordinator (JK) throughout the trial if needed.

##### Physiotherapy intervention

Physiotherapy interventions are detailed in Tables 1, 2 and Additional file 2. These consist of (i) manual hip joint and soft tissue mobilisation and stretching; (ii) hip muscle retraining; (iii) trunk muscle retraining; (iv) functional, proprioceptive and sports- or activity- specific retraining; (v) enhancing physical activity and (vi) education. The treatment will be tailored according to each patient’s clinical presentation (such as strength, pain severity, sporting and functional needs), the presence of co-morbidities (such as back and other lower limb pain or pathology), and progressed based on response to exercise load, thus maximising the training effects. The physiotherapist will supervise exercises during each visit. A home exercise program will be performed independently at home four times per week. An exercise manual will be made accessible to each participant.

**Table 1:** **Manual therapy techniques: a semi-standardised approach**

**Manual therapy techniques**				
**Technique**	**Aim**	**Description**	**Dosage**	**Timeframe**
Soft tissue massage and trigger point release of iliopsoas, adductor group, gluteus minimus, gluteus medius, piriformis and tensor fascia latae	Address soft tissue restrictions with the aim of reducing pain and increasing hip joint range of movement	Sustained digital pressure to each trigger point, with the muscle positioned on stretch	30 - 60 seconds digital pressure per trigger point	Session 1 - 8
		Massage longitudinally along the muscle belly	2 - 5 minutes of massage per muscle	
Mobilisation of lumbar spine	To improve lumbar spine mobility and restore normal lumbo-pelvic movement	Unilateral postero-anterior accessory glides, Grade III or IV	3 - 5 sets of 30 - 60 seconds	Session 1 - 8
Correction of sacro-iliac joint asymmetries	To optimise the position of the ilium and therefore the orientation of the acetabulum	Massage to iliopsoas	2 - 5 minutes of massage	Session 1 - 8
		Mobilisation of sacrum		
Manual traction if ligamentum teres is intact or ligated and patient is >3 months post-labral repair	Increase hip flexion and/or IR/ER range of motion	Seatbelt around patient's proximal femur and therapist's hips. Gentle inferior and/or lateral traction force applied. May include patient actively moving hip into flexion as traction is applied	3 sets of 10 seconds. If tolerated, increase by 1 set per treatment session to a maximum of 6 sets in total	Session 1 - 8

Legend: IR = internal rotation; ER = external rotation.

**Table 2:** **Home exercise program for hip muscle retraining, trunk muscle retraining, functional and activity specific retraining and stretching: a semi-standardised approach**

**Home exercise program**			
**Exercise**	**Aim**	**Description**	**Timeframe**
Deep hip rotator strengthening	Optimize hip neuromuscular control and improve dynamic stability of hip	See Additional file 2	Session 1 - 4
Hip extensor muscle strengthening	Optimize hip neuromuscular control and improve dynamic stability of hip	See Additional file 2	Session 1 - 6
Hip abductor muscle strengthening	Optimize hip neuromuscular control and improve dynamic stability of hip	See Additional file 2	Session 2 - 8
Functional strengthening	Improve gluteal and lower limb strength. Practice movement patterns required for optimal daily function	See Additional file 2	Session 3 - 8
Balance exercises	Improve proprioception and dynamic stability of hip and pelvis	See Additional file 2	Session 2 - 8
Anterior hip stretch	Assist in regaining full hip extension range of movement	See Additional file 2	Session 2 - 8

**Figure 2:**
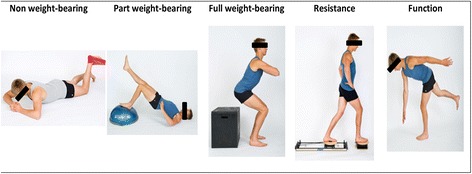
**Examples of hip exercise progression from non-weight bearing to functional tasks.**

Specific aspects of the treatment include:

1. Manual hip joint and soft tissue mobilisation and stretching to provide optimal joint range and facilitate control of movement patterns. Within each treatment session, the therapist will measure range of motion (with an inclinometer), and monitor the immediate effects of treatment modalities.
2. Hip muscle retraining, including exercise to improve hip abduction, extension and external rotator coordination and strength. In order to accommodate a heterogeneous cohort, the hip muscle retraining may be performed statically and/or dynamically in various functional activities (for example, step up and down, squat and/or sit to stand). Resistance will be progressed based on individual responses, and is detailed in Figure 2.
3. Trunk muscle retraining to improve strength, endurance and control of the trunk muscles. The exercise selection and progression will follow similar principles to the hip muscle retraining.
4. Functional, proprioceptive and sports-specific retraining. The exercise selection and progression will follow similar principles to the hip and trunk muscle retraining. For example, a person aiming to return to football may perform single leg activities with direction change and pain-free, graduated return to kicking, whilst maintaining good movement control.

##### Control

In order to control for the psychosocial contact inherent with the physiotherapy treatment, the control treatment will encompass individualised health education sessions covering topics such as exercise, diet, weight loss and appropriate stretching, in a similar fashion to previously published clinical trials looking at physiotherapy management in older people with advanced hip OA [39,40]. The information sheets have been modified to be appropriate for this younger age group. The control sessions will be provided with the same frequency and duration as the physiotherapy sessions.

##### Data management, monitoring and statistical analyses

Treatment efficacy will be evaluated by comparing change on primary outcome measures between groups. Baseline characteristics of participating patients of the two groups will be evaluated for their influence on outcomes and included as covariates in statistical analyses if required. Outcomes of interest will be analysed on an intention-to-treat basis for all participants. SPSS software (Version 21, SPSS Inc, Chicago, IL, USA) will be used for all analyses, and significance will be set at *P* <0.05. Data monitoring will be conducted by an independent investigator (KC), and data will be stored in a locked cabinet and password-secured server. Adverse events will be recorded by the treating physiotherapists who will inform the project coordinator (JK). Trial results will be made available to participants and will be published in a peer-reviewed journal.

### Consent

Written informed consent was obtained from the patients for publication of this manuscript and accompanying images. A copy of the written consent is available for review by the Editor-in-Chief of this journal.

## Discussion

This study provides detail of the protocol for a phase II RCT investigating the efficacy of a physiotherapy intervention for early-onset hip OA [24]. This phase II study will identify the willingness of patients to enter a phase III RCT, adherence to the interventions and possible drop-out rates. In addition, this study will provide information regarding the treatment effects sizes of the semi-individualised physiotherapy intervention described, and assist in power calculations which will inform future larger scale RCTs.

The physiotherapy intervention described herein is semi-individualised, wherein a standardised program is tailored to the individual patient’s needs, based on assessment and reassessment undertaken at each physiotherapy session. Impairments in hip range of motion and hip muscle strength have been identified previously in people with chondropathy of the hip [41]. Therefore we have included therapeutic interventions targeted to address these impairments in the physiotherapy intervention.

The control intervention is an alternative physiotherapy intervention, focussing on education and guidance, rather than a ‘wait and see’ control group. This will control for both the attention and advice provided by physiotherapists during individual treatment sessions and may facilitate recruitment, since all patients will receive an active intervention regardless of treatment allocation.

The findings of this study will provide guidance regarding the feasibility of a full-scale phase III RCT, prior to its undertaking [24]. It will also provide pilot data on the efficacy of the physiotherapy intervention described herein.

## Trial status

This trial is ongoing. At the time of submission of this protocol, 17 patients had been recruited into the study over a four-month recruitment period, and 16 patients had completed the follow-up period. There have been no reported adverse events, drop-outs or patients lost to follow-up to date.

## Additional files

Additional file 1:
**A randomized clinical trial of physiotherapy intervention for early-onset hip osteoarthritis.**
Additional file 2:
**Specific examples of exercises included in home exercise program.**

## Abbreviations

HOOS: Hip disability and osteoarthritis outcome score
ICC: Intraclass correlation coefficient
iHOT: International Hip Outcome tool
MCIC: Minimal clinically important change
N: Newton
OA: Osteoarthritis
RCT: Randomised controlled trial
SEM: Standard error of measurement
VAS: Visual analogue scale
WOMAC: Western Ontario MacMaster Universities Osteoarthritis Index
