# Supplementary material for: A phase II trial for the efficacy of physiotherapy intervention for early-onset hip osteoarthritis: study protocol for a randomised controlled trial
Source: Trials. 2015 Jan 27;16:26. doi: 10.1186/s13063-014-0543-7 (PMC4318367; doi:10.1186/s13063-014-0543-7)
Supplement: Additional file 1: — A randomized clinical trial of physiotherapy intervention for early-onset hip osteoarthritis. [file 13063_2014_543_MOESM1_ESM.docx]

A randomized clinical trial of physiotherapy intervention for early-onset hip osteoarthritis

**School of Health and Rehabilitation Sciences**

# Plain Language Statement

We invite you to participate in our research project “A randomized clinical trial of physiotherapy intervention for early-onset hip osteoarthritis”. We would like to give you some background information on why we think this project is important and on what we would like you to do if you decide to join us in this research.

**What is the purpose of this study?**

Hip osteoarthritis (OA) is associated with significant personal and societal burden, which is rising with increased life expectancy. Early hip OA is commonly seen during hip arthroscopic surgery, and can affect younger adults (age<55 years) with peak work and family commitments. Whilst hip arthroscopy is commonly undertaken for pain associated with early hip OA, no studies have examined best practice for post-operative rehabilitation. An effective intervention for early-onset hip OA will potentially have great personal and societal impact. The primary aim of this study is to establish the efficacy of physiotherapy intervention on hip-related symptoms in early-onset hip OA following hip arthroscopy.

**Who can participate in this study?**

You can participate in this study if you are aged between 18 and 50 years of age, and have undergone hip arthroscopy surgery in the previous 12 to 36 months.

You are **not** eligible to participate in this study if you cannot understand spoken or written English. In order to undertake the physical testing, you are **not** eligible if you have current back or lower limb pain or injury, or if you cannot walk unaided.

**What does the project involve?**

You will be invited to attend a physiotherapy clinic in Hobart to undergo an assessment to ensure you are eligible for the study. If eligible, you will remain at the physiotherapy clinic to undergo testing of your physical function. This will take approximately one hour of your time and you will be asked to wear shorts (provided). The tests conducted in the physical examination may include measures of hip joint motion, strength of the muscles around the hip, balance standing on a force platform and functional tasks such as stepping onto a step. Following the assessment, you will be asked via email to complete several questionnaires asking you about your pain, other hip-related symptoms and your levels of physical activity.

After the first assessment and completion of the questionnaires, you will be randomly allocated to one of the treatment groups. You will have an equal chance of receiving either of the two treatments. The person who is doing the testing will not know which treatment you will receive. You will then be required to attend the same physiotherapy clinic for 8 treatments over a 3 month period. This will consist of treatment once a week for four weeks and then once a fortnight for the remaining eight weeks. Each treatment will last 30 minutes and will be performed by an experienced physiotherapist. Both treatments are used regularly by physiotherapists, and have been shown to reduce pain in older adults with more established hip OA. You may be given a home exercise program to complete which will take approximately 15 minutes daily to perform. All treatments will be provided at no cost to you. The research group will access your past medical and surgical records to obtain details of your surgery and any pre-operative scan results.

You will then complete the same assessment 3 months later at the conclusion of the study as you completed at the start of the study, which will take approximately one hour of your time. We will also contact you in the subsequent years to check on your progress, and also possibly to attend the physiotherapy clinic to complete additional questionnaires and physical measures. We may also access your medical and surgical records relating to your hip arthroscopy.

You will not receive any payment for your participation, however you may ask for a copy of your assessment results.

**Are there any potential side effects?**

The physical tests represent usual examination by a physiotherapist. You may experience a small amount of discomfort in the joints or muscles during the physical examination. Please report to the researcher any undue discomfort or pain experienced during the testing. If the pain or discomfort is deemed to be excessive by yourself or the investigators, then testing will cease.

If required, emergency procedures will be used to deal with any medical event that arises during the testing. The physiotherapy clinics have documented procedures for emergencies. This includes annual St John’s ambulance CPR training and appropriate management of fire for all staff.

**What if I have any concerns during the study?**

This study adheres to the Guidelines of the ethical review process of The University of Queensland and the National Statement on Ethical Conduct in Human Research. Whilst you are free to discuss your participation in this study with project staff (contactable on 07 3365 3008), if you would like to speak to an officer of the University not involved in the study, you may contact the Ethics Coordinator on 07 3365 3924.

**Can I withdraw from the study if I wish?**

Your participation in this study is voluntary. If you do not wish to take part you are under no obligation to do so. Also, if you decide to take part but later change your mind, you are free to withdraw from the project at any stage. You may also withdraw any unprocessed data previously supplied by you.

If you are a patient of any of the investigators, your decision whether to take part or not to take part, or to withdraw, will **not** affect your management in any way.

If you are a staff member at the University, or of any of the investigators, your decision whether to take part or not to take part, or to withdraw, will **not** affect your future employment in any way.

If you are a student at the University, your decision whether to take part or not to take part, or to withdraw, will **not** affect your future grades or assessment in any way.

**Will my details be kept confidential?**

The anonymity of your participation is assured by our procedure, in which a code number and not your name will identify you. No findings that could identify you will be published and access to individual results is restricted to the investigators. Coded data will be stored for at least 5 years. All data and results will be handled in a strictly confidential manner, under guidelines set out by the National Health and Medical Research Council. The chief investigator is responsible for maintaining this confidentiality. This project is subject to the requirements of the Medical Research Ethics Committee of the University of Queensland. However, you must be aware that there are legal limitations to data confidentiality.

**How do I get more information?**

You should ask for any information you want. If you would like more information about the study, or if there is any matter about it that concerns you, either now or in the future, do not hesitate to ask one of the researchers. Before deciding whether or not to take part you may wish to discuss the matter with a relative or friend or with your local doctor. You should feel free to do this.

**About the researchers:**

**Dr Kay Crossley** is a sports physiotherapist and principal research fellow in the School of Health and Rehabilitation Sciences at the University of Queensland

**Dr Trevor Russell** is a physiotherapist and principal research fellow in the School of Health and Rehabilitation Sciences at the University of Queensland

**Dr Joanne Kemp** is a sports physiotherapist and a post-doctoral researcher at Federation University Australia.

**Kate Young** is a physiotherapist based in Hobart.

**Mr Michael Pritchard** is an Orthopaedic Surgeon specializing in arthroscopic surgery in Hobart

**To contact any of the researchers, please phone 07 3365 3008 or 03 6231 5991**

**THE UNIVERSITY OF QUEENSLAND**

School of Health and Rehabilitation Sciences

**Consent form for persons participating in research projects**

**Name of participant:**

Project title: A randomized clinical trial of physiotherapy intervention for early-onset hip osteoarthritis

**Investigator(s):** Dr Kay Crossley, Dr Joanne Kemp, Ms Kate Young, Mr Michael Pritchard

1. I consent to participate in the above project, the particulars of which - including details of tests or procedures - have been explained to me to my satisfaction.

1. I authorize the investigator or his or her assistant to use with me the tests, which includes procedures referred to under (1) above
2. I acknowledge that:

(a) The possible effects of the tests or procedures have been explained to me to my satisfaction;

(b) My participation is voluntary and I have been informed that I am free to withdraw from the project at any time and to withdraw any unprocessed data previously supplied;

(c) I will not gain financial benefit from participation;

(d) I have been informed that the confidentiality of the information I provide will be safeguarded subject to any legal requirements.

1. My participation or non-participation will not affect my ongoing medical management, my grades, nor employment status at The University of Queensland

I, ___________________________________________ consent to participate in the above project.

Signature Date

(Participant)

Signature Date

(Witness)
