# Supplementary material for: A phase II trial for the efficacy of physiotherapy intervention for early-onset hip osteoarthritis: study protocol for a randomised controlled trial
Source: Trials. 2015 Jan 27;16:26. doi: 10.1186/s13063-014-0543-7 (PMC4318367; doi:10.1186/s13063-014-0543-7)
Supplement: Additional file 2: — Specific examples of exercises included in home exercise program. [file 13063_2014_543_MOESM2_ESM.docx]

**Additional file 2:** Specific examples of exercises included in home exercise program

| Exercise Aim | Dosage | Exercise Options |  |  |  |
| --- | --- | --- | --- | --- | --- |
| Deep hip rotator strengthening | 2 - 3 sets | 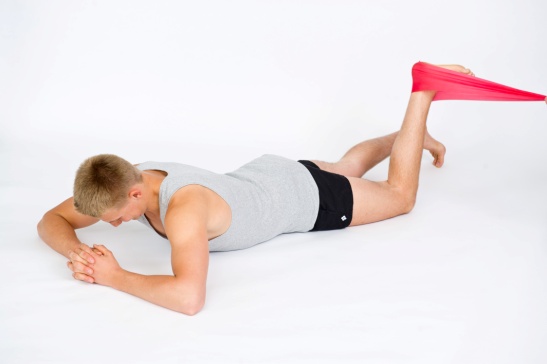 | 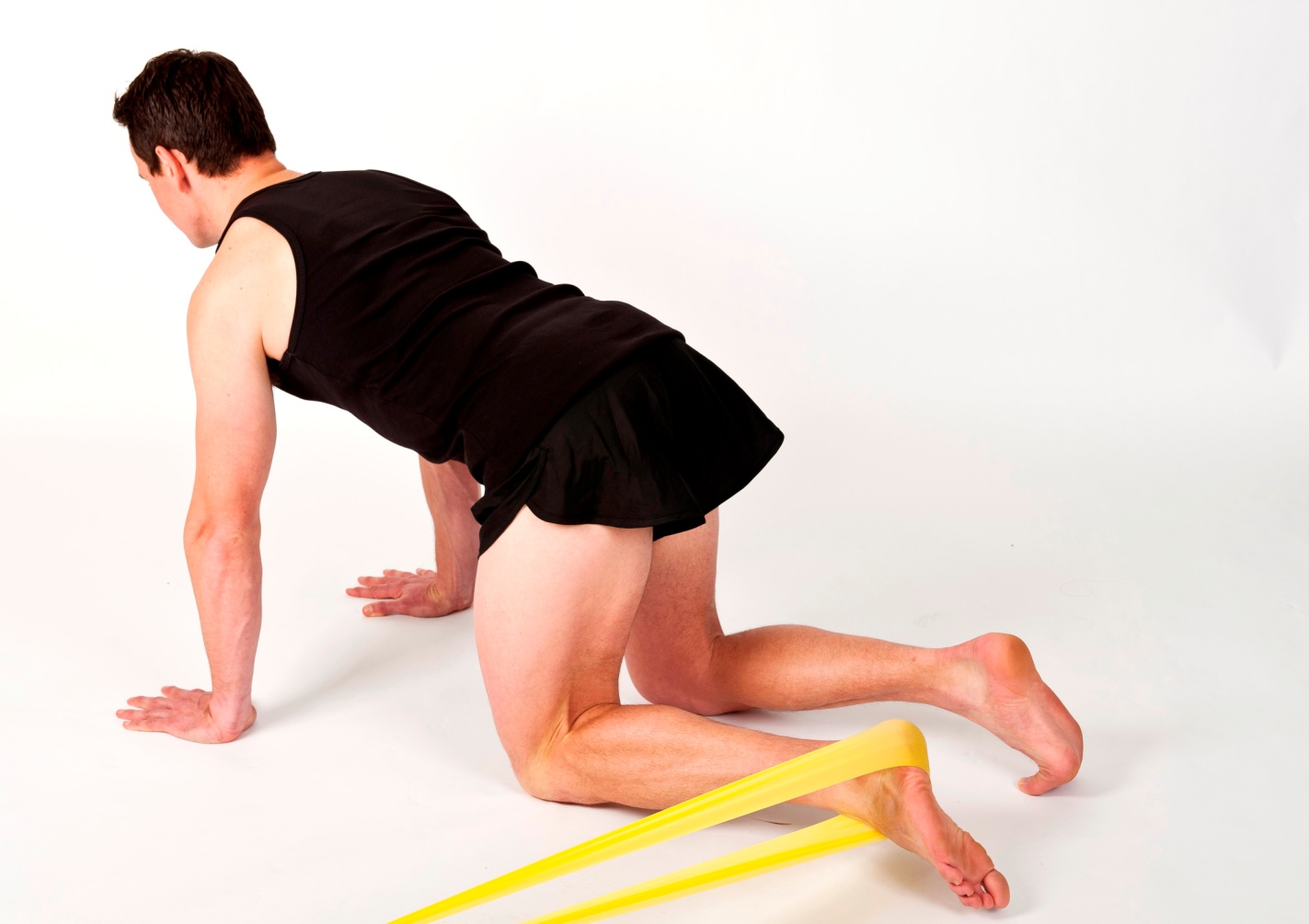 |  |  |
|  | 20 - 40 reps | Prone Hip ER | Four Point Kneel Hip ER |  |  |
|  | 1 - 3 times per day | Start: isometric 5-second hold against band | Start: isometric 5-second hold against band |  |  |
|  |  | Progress: concentric-eccentric through range hip ER | Progress: concentric-eccentric through range hip ER |  |  |
| Hip extensor muscle strengthening | Motor control: | 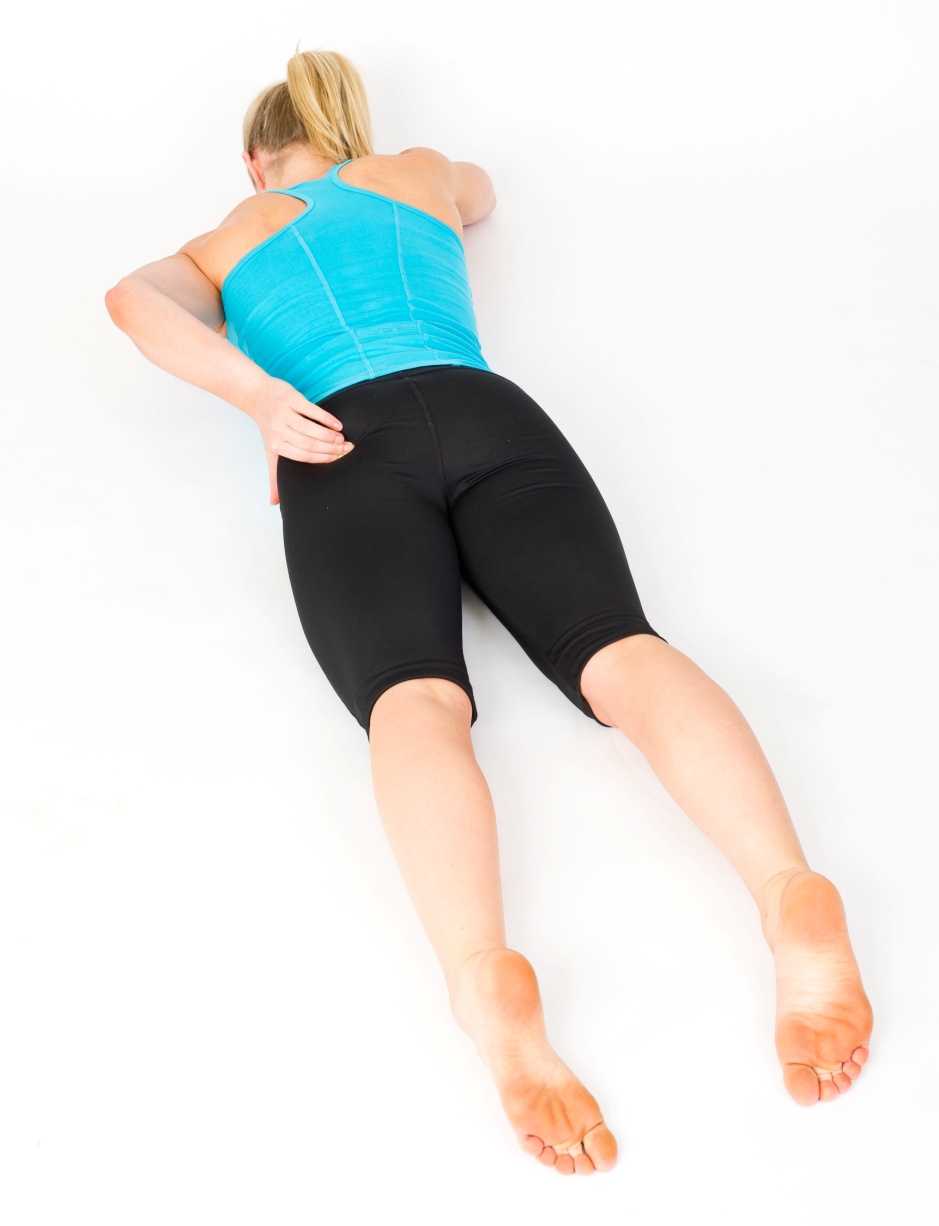 | 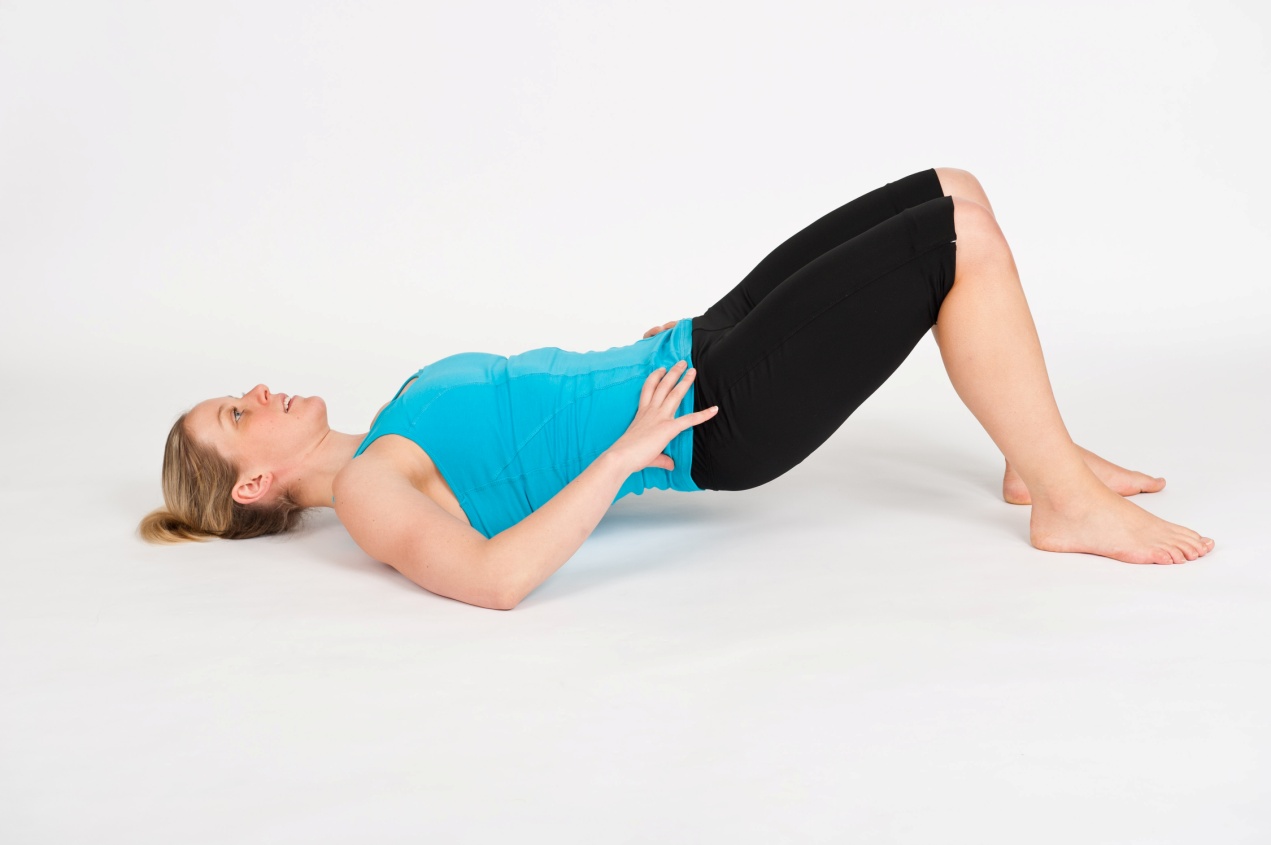 | 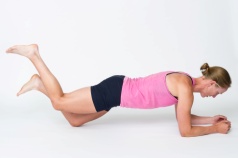 | 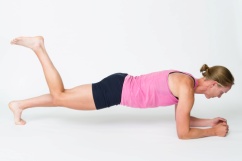 |
|  | 2 - 3 sets | Prone Hip Extension | Bridging | Prone Hold Hip Extension - knees | Prone Hold Hip Extension - toes |
|  | 20 - 40 reps | Gluteal squeeze and leg extension 3 - 5-second hold and lower | Gluteal squeeze and lift up into bridge position 3 - 5-second hold and lower | From knees move affected leg into hip extension 3 - 5-second hold and lower leg | From toes move affected leg into hip extension 1 - 5-second hold and lower leg |
|  | 1 - 3 times per day |  |  |  |  |
|  | Strength: |  |  |  |  |
|  | 1 - 3 sets |  |  |  |  |
|  | 6 - 12 reps |  |  |  |  |
|  | Once daily |  |  |  |  |
| Hip abductor muscle strengthening | Motor control: |  |  |  |  |
|  | 2 - 3 sets | 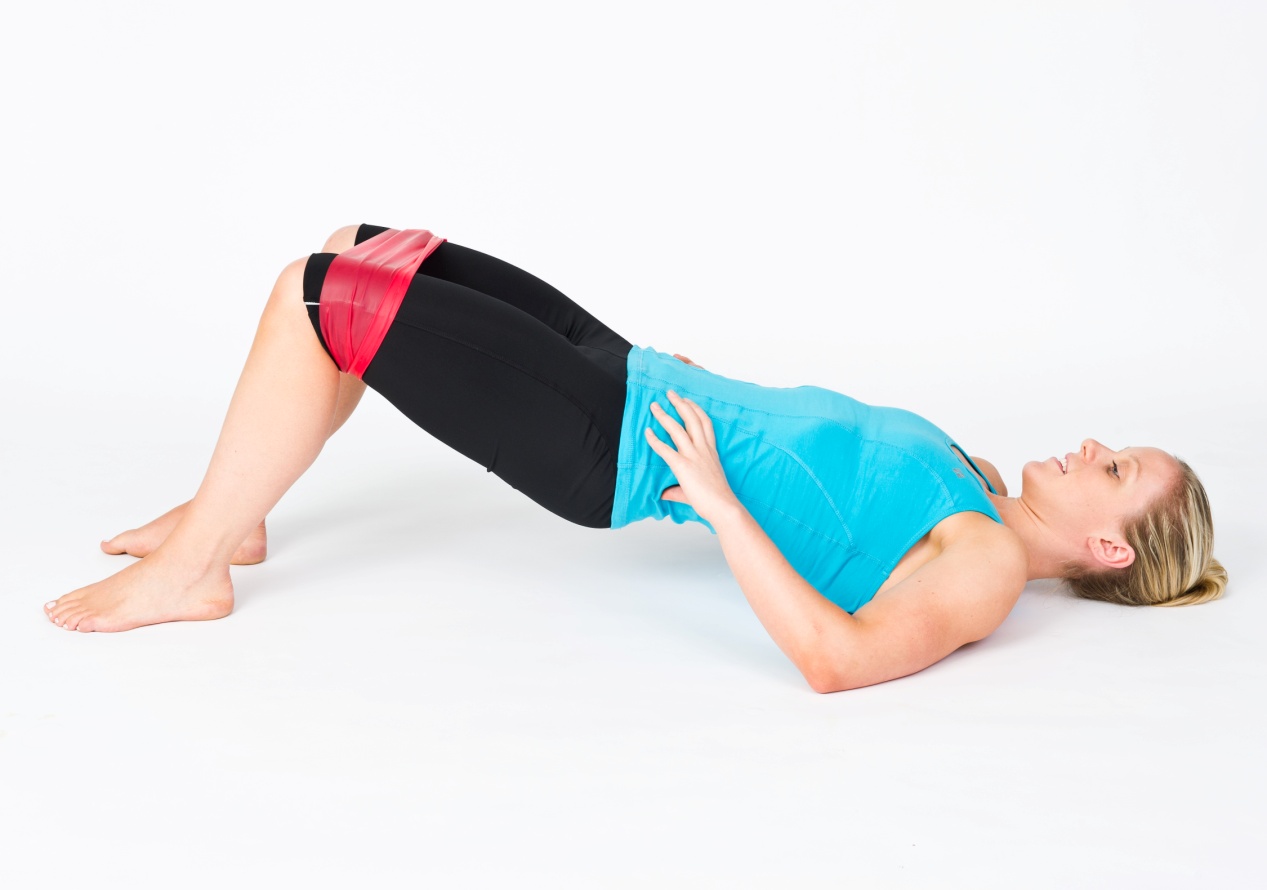 | 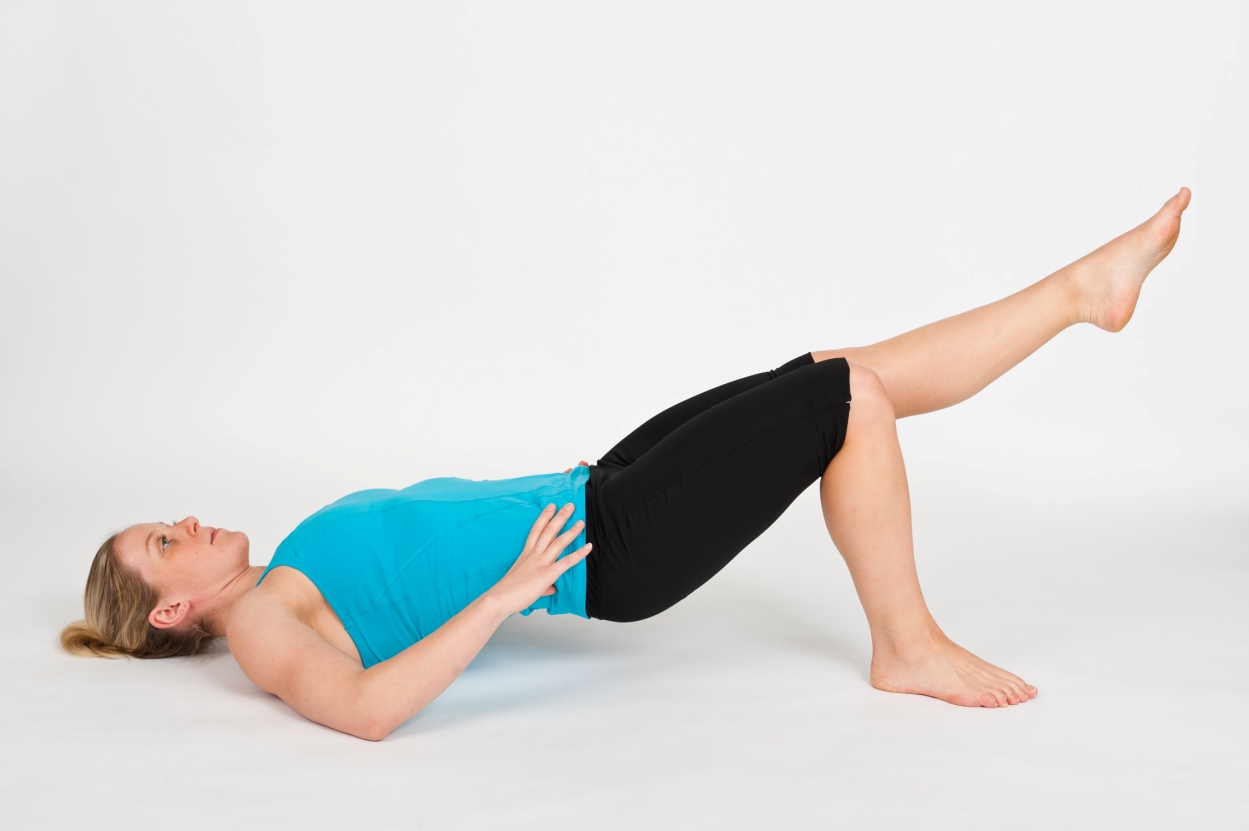 | 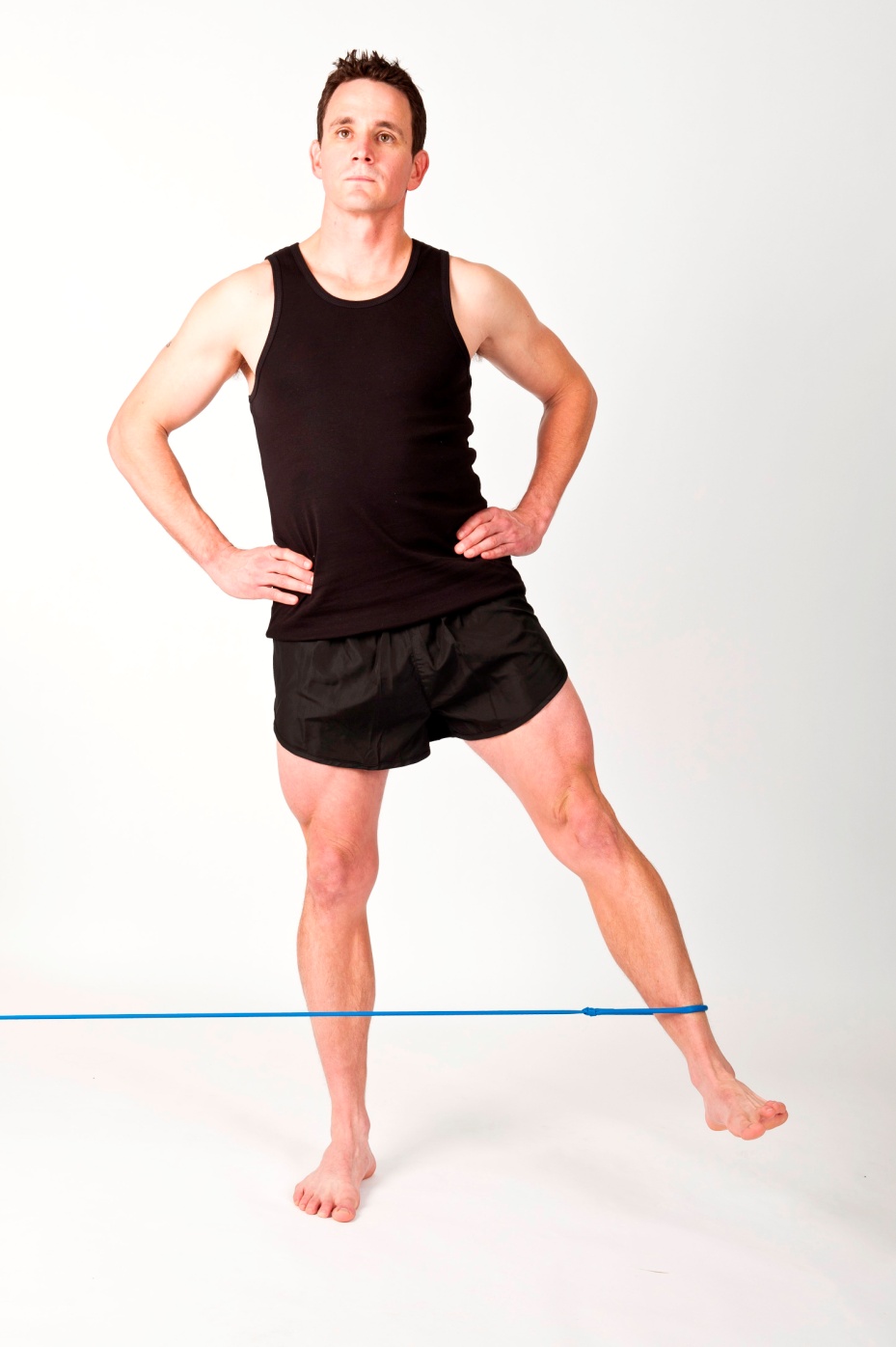 | 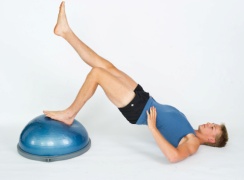 |
|  | 20 - 40 reps | Bridging with Band | Bridge with Leg Extension | Standing Hip Abduction | Bridge with Foot on Unstable Surface |
|  | 1 - 3 times per day | Bridge with band around knees, gently abduct against band. | Start: lift up with two feet on ground, extend one leg then the other then lower with both legs on ground. | Single leg stance and abduct leg out to side. Keep trunk straight, do not laterally flex. | Keep pelvis level, activate gluteals and lift up |
|  | Strength: |  |  |  |  |
|  | 1 - 3 sets | 3 - 5-second hold and lower | Progression: extend unaffected knee, lift up using affected side, 2 - 5-second hold and lower | Perform both standing and then with band on affected side |  |
|  | 6 - 12 reps |  |  |  |  |
|  | Once daily |  |  |  |  |
| Functional strengthening | 2 - 3 sets | 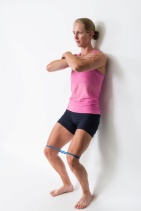 | 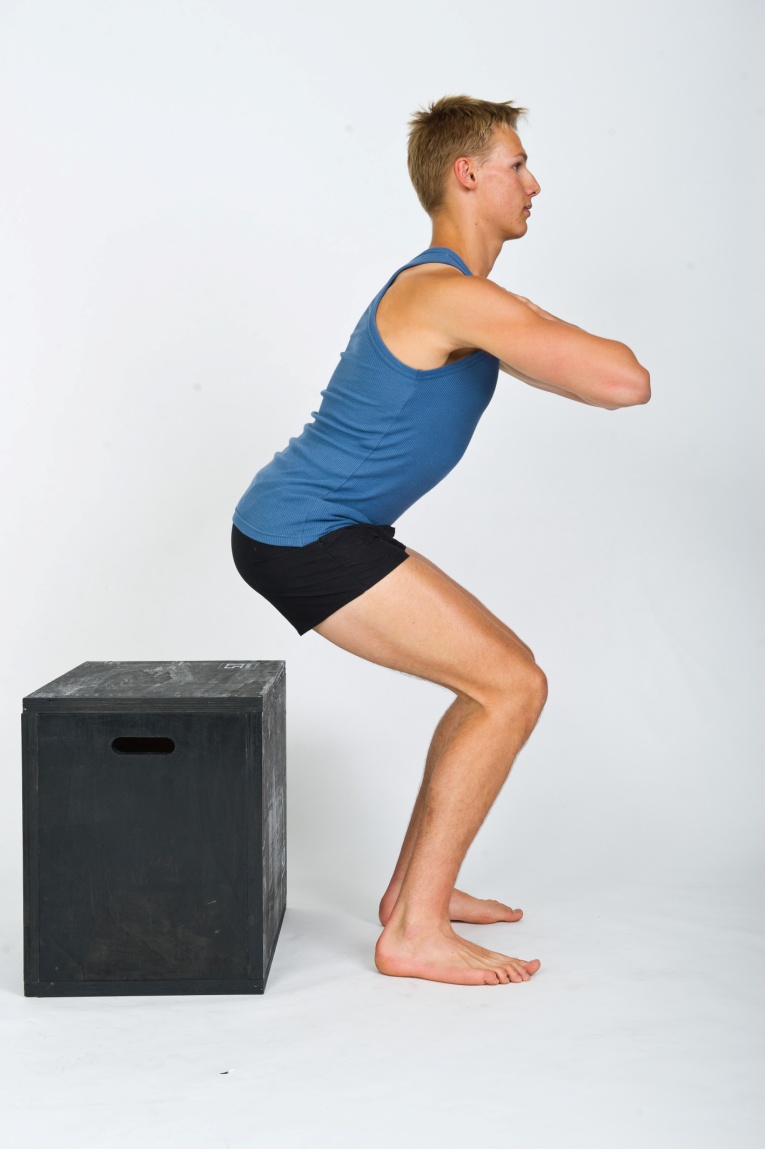 | 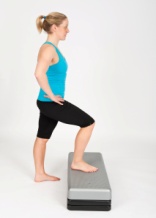 | 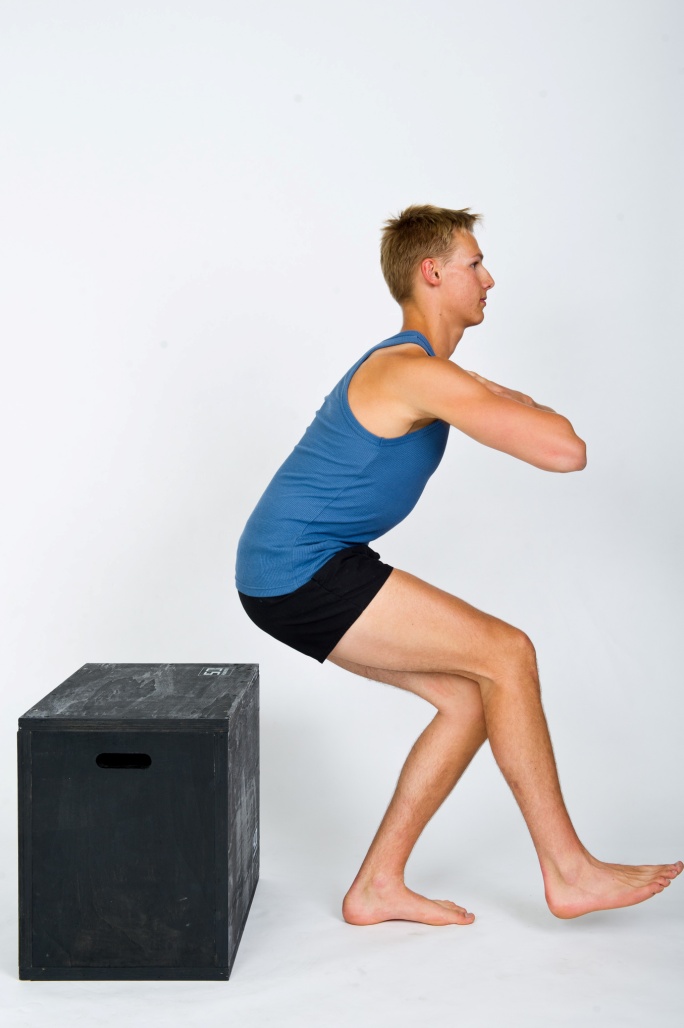 |
|  | 15 - 20 reps |  |  |  |  |
|  | Once daily | Wall Slides with Gluteal Activation | Squats | Step Ups | Single Leg Squats |
|  |  | Band around distal thighs. Slide down wall, activate gluteal muscles at 60 - 90° knee flexion, 5 - 30-second hold then push back up into standing | Flex at hips and squat to comfortable depth, tighten gluteal muscles to return to standing | Affected side on top of step, tighten gluteal muscles to step unaffected side up onto step | Stand on affected side, squat down to comfortable level ensuring adequate hip, knee and ankle alignment. Tighten gluteals to return to standing |
| Balance exercises | 1 - 3 minutes | 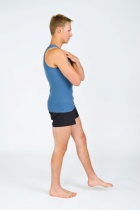 | 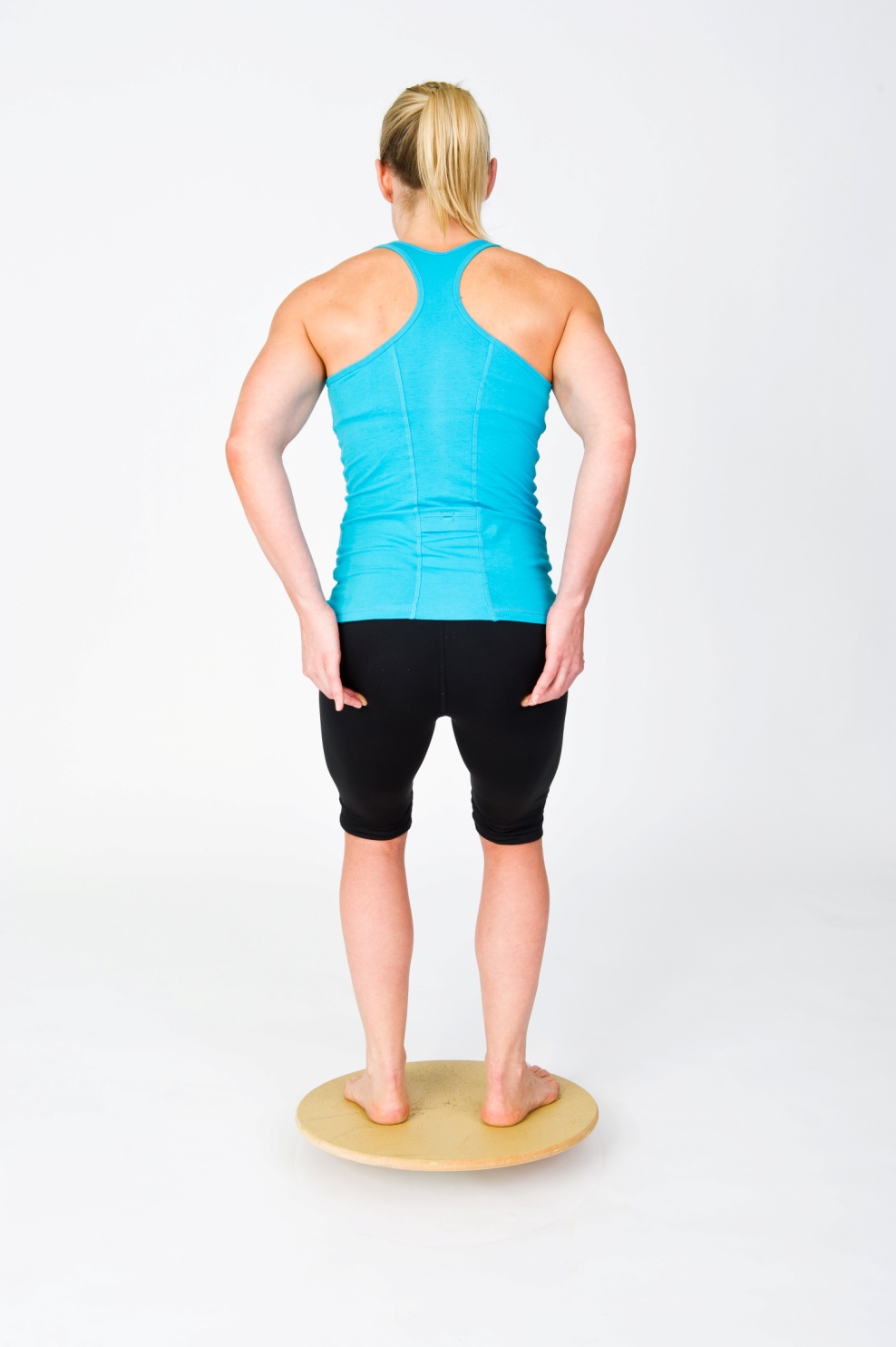 | 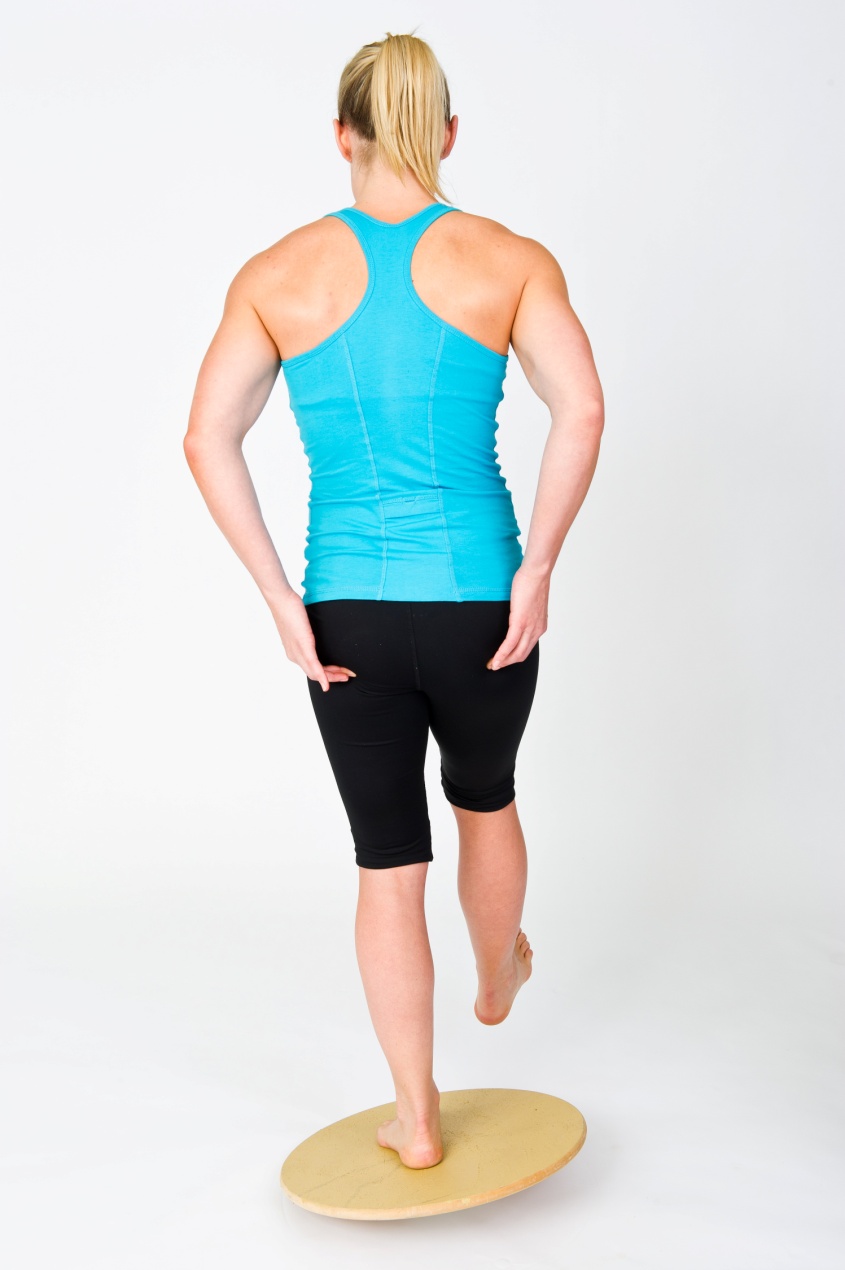 | 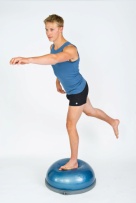 |
|  | daily | Single Leg Balancing | Wobble Board with Gluteal Activation | Wobble Board with Gluteal Activation - Single leg | Gluteal Activation with Functional Movements |
|  |  | Stand on affected leg, ensuring no Trendelenburg sign | Balance on wobble board maintaining gluteal activation | Single leg balance on wobble board, maintaining gluteal activation | Maintain gluteal activation on unstable surface, lean trunk forward and reach with contralateral upper limb |
| Anterior hip stretch | 1 - 2 minutes | 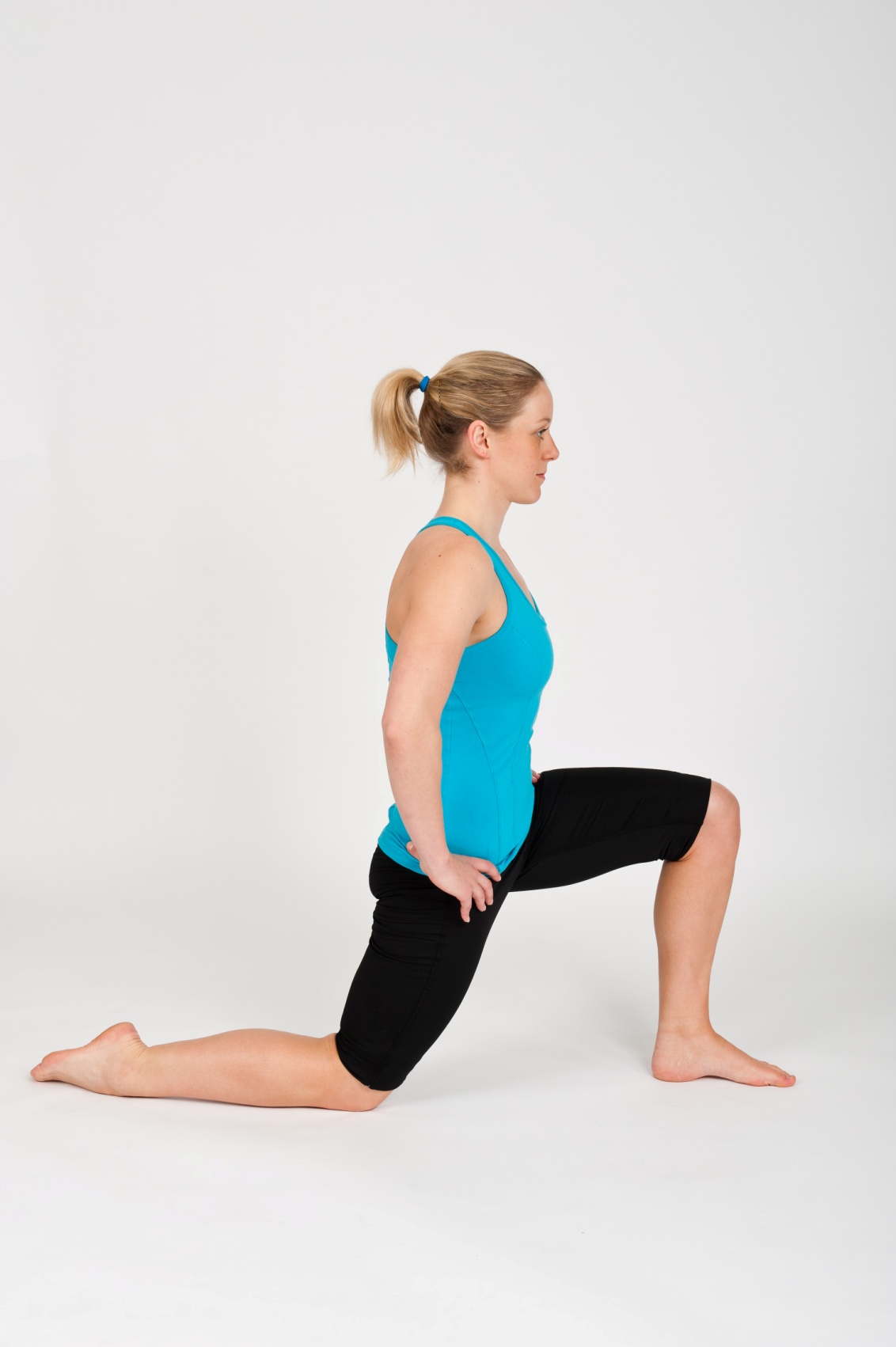 |  |  |  |
|  | 1 - 3 times daily | Anterior Hip Stretch |  |  |  |
|  |  | Ensure pelvis is posteriorly tilted, trunk upright and gluteals activated on back leg |  |  |  |

Legend: ER = external rotation; IR = internal rotation
